# Postoperative morbidity and health-related quality of life in children with delayed reconstruction of esophageal atresia: a nationwide Swedish study

**DOI:** 10.1186/s13023-022-02381-y

**Published:** 2022-06-20

**Authors:** Michaela Dellenmark-Blom, Sofie Örnö Ax, Elin Öst, Jan F. Svensson, Ann-Marie Kassa, Linus Jönsson, Kate Abrahamsson, Vladimir Gatzinsky, Pernilla Stenström, AnnaMaria Tollne, Erik Omling, Helene Engstrand Lilja

**Affiliations:** 1 Department of Pediatrics, Institute of Clinical Sciences, The Queen Silvia Children’s Hospital, Gothenburg University, 416 85 Gothenburg, Sweden; 2 Department of Pediatric Surgery, Queen Silvia Children’s Hospital, Sahlgrenska University Hospital, Gothenburg, Sweden; 3 Department of Pediatric Surgery, Karolinska University Hospital, Stockholm, Sweden; 4 Department of Women’s and Children’s Health, Karolinska Institutet, Stockholm, Sweden; 5 Department of Women’s and Children’s Health, Uppsala University, Uppsala, Sweden; 6 Department of Pediatric Surgery, University Children’s Hospital, Uppsala, Sweden; 7 Department of Pediatric Surgery, Skane University Hospital, Lund University, Lund, Sweden

**Keywords:** Esophageal atresia, Long-gap esophageal atresia, Health-related quality of life, Delayed reconstruction, Long-term morbidity, Postoperative outcomes

## Abstract

**Background:**

In 10–15% of children with esophageal atresia (EA) delayed reconstruction of esophageal atresia (DREA) is necessary due to long-gap EA and/or prematurity/low birth weight. They represent a patient subgroup with high risk of complications. We aimed to evaluate postoperative morbidity and health-related quality of life (HRQOL) in a Swedish national cohort of children with DREA.

**Methods:**

Postoperative morbidity, age-specific generic HRQOL (PedsQL^™^ 4.0) and condition-specific HRQOL (The EA-QOL questionnaires) in children with DREA were compared with children with EA who had primary anastomosis (PA). Factors associated with the DREA group’s HRQOL scores were analyzed using Mann–Whitney U-test and Spearman’s rho. Clinical data was extracted from the medical records. Significance level was *p* < 0.05.

**Results:**

Thirty-four out of 45 families of children with DREA were included and 30 returned the questionnaires(n = 8 children aged 2–7 years; n = 22 children aged 8–18 years). Compared to children with PA(42 children aged 2–7 years; 64 children aged 8–18 years), there were no significant differences in most early postoperative complications. At follow-up, symptom prevalence in children aged 2–7 with DREA ranged from 37.5% (heartburn) to 75% (cough). Further digestive and respiratory symptoms were present in ≥ 50%. In children aged 8–18, it ranged from 14.3% (vomiting) to 40.9% (cough), with other digestive and airway symptoms present in 19.0–27.3%. Except for chest tightness (2–7 years), there were no significant differences in symptom prevalence between children with DREA and PA, nor between their generic or condition-specific HRQOL scores (*p* > 0.05). More children with DREA underwent esophageal dilatations (both age groups), gastrostomy feeding (2–7 years), and antireflux treatment (8–18 years), *p* < 0.05. Days to hospital discharge after EA repair and a number of associated anomalies showed a strong negative correlation with HRQOL scores (2–7 years). Presence of cough, airway infection, swallowing difficulties and heartburn were associated with lower HRQOL scores (8–18 years), *p* < 0.05.

**Conclusions:**

Although children with DREA need more treatments, they are not a risk group for postoperative morbidity and impaired HRQOL compared with children with PA. However, those with a long initial hospital stay, several associated anomalies and digestive or respiratory symptoms risk worse HRQOL. This is important information for clinical practice, families and patient stakeholders.

**Supplementary Information:**

The online version contains supplementary material available at 10.1186/s13023-022-02381-y.

## Background

Esophageal atresia (EA) with or without a tracheoesophageal fistula (TEF) remains rare, with a prevalence of 2.4 in 10,000 live births [1]. Nevertheless, primary anastomosis (PA) of EA with distal TEF has become a standard procedure with over 90% survival rates [2]. In 10–15% of cases, the reconstruction of EA is delayed, because the gap between the two esophageal ends is too long (long-gap EA, LGEA) [3, 4], or related to the neonate’s prematurity/birth weight [5–7]. Children with delayed reconstruction of EA (DREA) represent a patient group with a high risk of future morbidity [3, 8–10].

Historically, LGEA is managed by inserting a gastrostomy for enteral feeding, allowing for spontaneous growth of the esophageal segments, then performing a delayed primary anastomosis (DPA) when the child is 3- 4 months old [8]. The native esophagus can also be preserved following elongation techniques, like Foker’s technique [11, 12] or Kimura’s advancement [13]. Esophageal replacement (ER) may also be employed using stomach, jejunum or colon and with the conduit of choice depending on the clinical center [3, 4, 14, 15]. When neonates with EA are extremely premature and/or have very low to extremely low birth weight, primary or staged repairs are used [5, 7, 16, 17].

Children with LGEA as opposed to short-gap EA more commonly present with cardio-vascular malformations [18, 19], genetic disorders and prematurity/low birth weight [18]. Moreover, both children with LGEA and premature children with EA are at higher risk of developing long-term gastrointestinal and respiratory sequelae [9, 15, 20–30].

Although health-related quality of life (HRQOL) [31] research in patients with EA has successively expanded, knowledge of HRQOL in children with DREA is scarce [32, 33]. One study demonstrated worse generic HRQOL in preschool children with isolated EA vs those with EA and distal TEF [34]. Other studies found similar HRQOL in EA children with jejunal interposition, gastric pull-up (GPU) [35] and thoracoscopic external traction technique [36] compared with healthy children. Likewise, patients with EA and esophagocoloplasty [37] and gastric tube interposition have good HRQOL [38]. Prematurity [34, 39] is associated with impaired generic HRQOL in children with EA. Recently, condition-specific HRQOL questionnaires for children with EA were developed (the “EA-QOL questionnaires”). Following their validation [40–42], these have not yet been applied specifically to children with DREA.

We aimed to evaluate postoperative morbidity and generic as well as condition-specific HRQOL in Swedish children with DREA aged 2–7 and 8–18 years, including DPA and ER, comparing them with children who underwent PA. Within the DREA group, also to identify factors associated with lower HRQOL scores and assess parent–child agreement in rating the child’s HRQOL.

## Methods

### Ethics

This study was approved by the Swedish Ethical Committee in 2019 (2019-04,930) and 2020 (2020-04,310).

### Setting

**Fig. 1:**
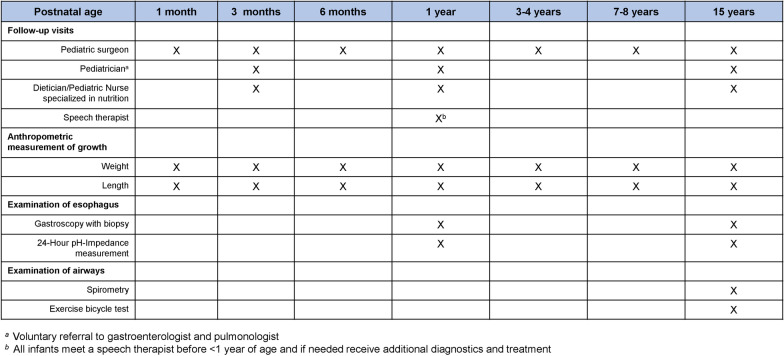
Presentation of the Swedish follow-up program at a tertiary pediatric surgical center for children born with esophageal atresia. The visits include a check-up and multidisciplinary monitoring of digestive and airway problems, growth, development of winged-scapula and scoliosis. At 1 and 15 years of age, patients are at a minimum offered physiological examination of the esophagus (e.g. 24-Hour pH-Impedance, gastroscopy with biopsy and at 15 years, test of the airways (e.g. spirometry) and exercise bicycle test

In Sweden, an average of 32 infants are annually born with EA [43]. Historically, these children have been surgically treated at four tertiary pediatric surgical centers. The children are offered standardized follow-up care at a tertiary pediatric surgical center (ie a minimum amount of follow-up with care inbetween as needed) according to a national follow-up program established in 2011 (Fig. 1).

### Study participants

Families of children with EA Gross type A (isolated EA), B (EA with proximal TEF), C (EA with distal TEF), Gross D (EA with proximal and distal TEF) were eligible for recruitment if the child was aged between 2 and 18 at the time of the study and they were fluent in written and spoken Swedish. Children aged < 8 years and children with cognitive dysfunction, were represented by their parent-proxy reports only. Children aged ≥ 15 years and legal guardians of children aged 2–18 years needed to give written informed consent to participate.

#### Children with DREA

Children were considered to have DREA when primary anastomosis was not achievable at the first operation either because it was too far between esophageal segments, or because of the degree of prematurity/birth weight, meaning that these children received a gastrostomy/jejunostomy for enteral feeding. Forty-five children with DREA were identified through hospital records from the Karolinska University Hospital, Stockholm (n = 15), the Uppsala University Hospital, Uppsala (n = 14), Sahlgrenska University Hospital, Gothenburg (n = 13) and Skåne University Hospital, Lund (n = 3). Their anatomical subtypes were Gross A (n = 19), Gross B (n = 12) and Gross C (n = 14) and they underwent esophageal reconstruction with DPA (n = 18), gastric tube esophagoplasty preserving the distal esophageal segment (n = 12), partial GPU (n = 6), GPU (n = 5) and colon interposition (n = 4). Of the families, two did not respond, one patient was deceased, one had moved abroad, one was excluded for social reasons, one for lack of skills in Swedish and five families declined to participate. Hence, the study included families of 34 children aged 2–18 with DREA (10 children aged 2–7 and 24 children aged 8–18).

#### Comparison group; children with Gross type C who underwent primary anastomosis.

The children with Gross type C who underwent PA were recruited from Sahlgrenska University Hospital, Gothenburg. They included 106 families (42 children aged 2–7; 64 children aged 8–18) who had participated in an earlier study of generic HRQOL [34] and/or the field test of the EA-QOL questionnaires [42] with ≥ 90% response rate, and served as a comparison group for children with DREA.

### Data collection

Families of children with DREA received questionnaires with pre-stamped reply envelopes to increase response rates, and non-respondents received a maximum of three reminders. Data was collected from mid-January to March in 2020, then was paused due to the covid-19 pandemic. The last four replies were collected between February and April in 2021.

#### Clinical data

A researcher at each center reviewed medical records for birth characteristics, Gross EA-type, initial gap length measured in centimeters or vertebral bodies as available, associated anomalies, surgical interventions, reasons for delayed reconstruction, postoperative outcomes, time to esophageal reconstruction and to hospital discharge from the tertiary care. Data on the child’s health, including presence of digestive and airway symptoms and medication intake the previous four weeks, were collected through a parent-reported questionnaire, which had also been used on children with PA [34, 42].

#### Parent characteristics

One parent of each child answered a survey asking for information about the participating parent, including parental age, marital status and educational level.

#### Generic HRQOL

Generic HRQOL was measured by PedsQL^™^ 4.0 generic core scales (PedsQL^™^ 4.0) which has been psychometrically evaluated for use in healthy children and children with chronic conditions. The PedsQL 4.0 for children aged 2–4 comprises 21 items, while the versions for children aged 5–7, 8–12 and 13–18 years include 23 items. The aspects measured are physical (8 items), emotional (5 items), social (5 items), and school functioning (5 or 3 items). Questions are answered using a 4-week recall period, using a 5-point Likert scale [44, 45].

#### Condition-specific HRQOL

Condition-specific HRQOL was measured by the EA-QOL questionnaires, which were originally developed and validated in Sweden and Germany [40–42]. The version for children aged 2–7 consists of 17 items, the domains being eating (7 items), physical health & treatment (6 items) and social isolation & stress (4 items). The version for children aged 8–18 consists of 24 items, the domains being eating (8 items), social relationships (7 items), body perception (5 items) and health & wellbeing (4 items). Questions were answered using a 4-week recall period, and a five-point Likert scale [40–42].

### Data analysis

Data were analyzed using IBM SPSS Statistics for Windows (version 25.0, Armonk, NY, USA: IBM Corp) and SAS 9.4(SAS Institute Inc., Cary, NC, USA). The 5-point Likert scale responses to the PedsQL^™^ 4.0 [44, 45] and the EA-QOL-questionnaires [42] were linearly transformed to a 0–100 scale, with higher levels denoting better HRQOL. We required ≥ 70% of item responses for scale score calculations. The children’s HRQOL scores were analyzed in age groups 2–7 (parent-report) and 8–18 (child-and-parent report respectively) in accordance with the instrument’s design [42] and age for child self-report (8 years). For continuous variables, median and range were calculated and for categorial variables, frequencies and percentages. Tests of significance included non-parametric methods. Mann–Whitney U-test and the Kruskal–Wallis H test respectively, were used to determine if there were statistically significant differences between two or more groups, when the dependent variable was ordinal or continous (and when the subgroups had ≥ 5 observations). Fisher's exact test was used to determine if there were associations between two categorical variables and Pearson Chi Square for more than two categorial variables. Spearman’s rank correlation (Spearman’s rho) was used for bivariate correlation analysis, with Spearman’s rho considered weak (0–0.39), moderate (0.40–0.59), strong (≥ 0.60). In children aged 8–18 with DREA, child-parent agreement in ratings of the child’s HRQOL were calculated using intra-class correlation coefficient (ICC) with values considered moderate (0.5–0.74), good (0.75–0.9) and excellent (> 0.90). Significance level was considered at p < 0.05.

## Results

### Study participants

Out of 34 families who accepted study participation, 30 families of children with DREA (n = 8 children aged 2–7 years; n = 22 children aged 8–17 years) gave informed consent and returned the questionnaires. Median age at follow-up was similar in children with DREA to children with PA, both in the younger group (6 years vs 5 years, p = 0.24) and in the older group (13 years vs 13 years, p = 0.68). In this study, subgroup analysis of 2–7-year-olds with DREA was not feasible due to low sample size.

### Congenital and parent-proxy characteristics

Table 1 presents the congenital/neonatal characteristics of children with DREA and children with PA and characteristics of the parent acting as proxy. Additional file 1  details the congenital/neonatal characteristics of children with DPA, ER and PA aged 8–18, characteristics of their parent-proxy, their postoperative morbidity and treatment at follow-up. In both children aged 2–7 and 8–18 with DREA, there were significant differences in congenital characteristics associated with disease severity compared to children with PA (p < 0.05), but no significant differences regarding parent characteristics (Table 1).

**Table 1﻿:** Congenital/neonatal and parent characteristics in children with delayed compared to primary reconstruction of esophageal atresia

	Children aged 2–7 years: n(%)					Children aged 8–18 years: n(%)				
*Child charateristic*s	n_tot_	Delayed reconstruction	n_tot_	Primary anastomosis	p-value	n_tot_	Delayed reconstruction	n_tot_	Primary anastomosis	p-value
Child gender male	8	5 (62.5)	42	27 (64.3)	1.0	22	10 (45.5)	64	34 (53.1)	0.62
Gestational age weeks, median (range)	8	36 (26–37)	42	37 (31–41)	0.096	22	36 (24–40)	62	38 (28–43)	**0.002**
Prematurely born (< 37 gestational weeks at birth)	8	5 (62.5)	42	19 (45.2)	0.46	22	13 (59.1)	62	17 (27.4)	**0.010**
Birth weight grams, median (range)	8	2202 (590- 2855)	41	2653 (1614–4260)	**0.014**	22	2312 (525–3225)	62	2720 (1070–3390)	**0.030**
Low birth weight (< 2500 g at birth)	8	7 (87.5)	41	21 (51.2)	0.12	22	13 (59.1)	62	18 (29.5)	**0.020**
Gross type esophageal atresia	8	A = 3 (37.5) B = 1 (12.5) C = 4 (50.0)	42	C = 42 (100)	–	22	A = 9( 40.9) B = 7( 31.8) C = 6 (27.3)	64	C = 64 (100)	**–**
Associated anomalies^a^	8	8 (100)	42	24 (57.1)	**0.039**	22	12 (54.5)	64	39 (60.9)	0.62
Cardiovascular	8	5 (62.5)	42	9 (21.4)	**0.030**	22	5 (22.7)	64	22 (34.4)	0.43
Anorectal	8	2 (25.0)	42	6 (14.3)	0.60	22	5 (22.7)	64	4 (6.3)	**0.044**
Urogenital	8	4 (50.0)	42	4 (9.5)	**0.016**	22	7 (31.8)	64	8 (12.5)	0.053
VACTERL association^b^	8	3 (37.5)	42	6 (14.3)	0.14	22	3 (13.6)	64	11 (17.2)	1.0
Verified genetic disorder	8	1 (12.5)	42	3(7.1)	0.51	22	4 (18.2)	64	7 (10.9)	0.46
*Reason for delayed reconstruction*										
Initital gap length, median cm (range)	3	4 (2–7)								
Initital gap length, median vertebral bodies (range)	3	4 (3.5–4)				19	4 (2–6)^c^			
Degree of prematurity, median (range)	2	29 (26–31)				3	29 (24–30)			
*Characteristics of parent acting as proxy*										
Parent respondent mother	8	5 (62.5)	42	37 (88.1)	0.11	22	18 (81.8)	63	56 (88.9)	0.46
Parental age median (range	8	40 (35–47)	42	37 (26–46)	0.09	22	46 (33–58)	63	44 (33–69)	0.26
Cohabitant partner	7	6 (85.7)	42	38 (90.5)	0.55	22	21 (91.5)	63	50 (79.4)	0.10
University/College education	8	4 (50.0)	42	23 (54.8)	1.0	22	13 (59.1)	63	31 (49.2)	0.47

Significant level was *p* < 0.05. Significant p-values are marked with bold text

^a^cardio-vascular, gastrointestinal, urogenital, limb, vertebrae-rib, choanalatresia, eye, ear, central nervous system or respiratory anomaly

^b^stands for vertebral defects, anal atresia, cardiac defects, tracheo-esophageal fistula, renal anomalies, and limb abnormalities. People diagnosed with VACTERL association have at least three of these characteristic features

^c^4 missing data in the presentation of initial gap length measured in vertebral bodies

### Initial surgical treatment

In the DREA group of children 2–7 years, two underwent DPA, three GPU and three gastric tube esophagoplasty preserving the distal esophagus. The reconstruction of EA took place at a median of 174 days (range 48–1221) and none had antireflux surgery at the time of esophageal reconstruction. In the DREA group aged 8–18 years, 12 children underwent DPA, five gastric tube esophagoplasty preserving the distal esophagus, three partial GPU and two colon interposition. Six children with ER had antireflux surgery at reconstruction. The delayed reconstruction took place at a median of 137 days (range 34–323), and there was no significant difference between DPA (median 113 days, range 34–323) and ER (median 164 days, range 36–314), p = 0.29.

### Postoperative morbidity

#### Early postoperative complications

Table 2 displays the postoperative course before discharge from a tertiary pediatric surgical ward for children with DREA vs children with PA. There were no significant differences between the groups for most of the complications assessed (Table 2).

**Table 2:** Postoperative course in children with delayed compared to primary reconstruction of esophageal atresia

	Children aged 2–7 years: n(%)						Children aged 8–18 years: n(%)			
	n_tot_	Delayed reconstruction	n_tot_	Primary anastomosis, Gross type C	p-value	n_tot_	Delayed reconstruction	n_tot_	Primary anastomosis, Gross type C	p-value
Revisional surgery after repair of EA eg due to anastomotic leakage or recurrent fistula	8	1 (12.5)	42	3 (7.1)	0.51	22	4 (18.2)	62	7 (10.9)	0.46
Anastomotic leakage	8	1 (12.5)	42	2 (4.8)	0.41	22	8 (36.4)	62	8 (12.9)	**0.026**
Sepsis verified through blood culture	8	4 (50.0)	42	8 (19.0)	0.082	22	6 (27.3)	62	6 (9.7)	0.071
Wound infection	8	1 (12.5)	42	1 (2.4)	0.30	22	1 (4.5)	62	2 (3.2)	1.0
Pneumothorax treated with drainage	8	3 (37.5)	42	2 (4.8)	**0.024**	22	3 (13.6)	62	11 (17.7)	0.75
Esophageal dilatation before hospital discharge	5	2 (20.0)	42	1 (2.4)	0.20	20	2 (10.0)	60	8 (13.3)	1.0
Days before discharge from tertiary pediatric surgical care, median (range)	8	230(69–1235)	42	31 (4–231)	** < 0.0001**	22	188 (62–364)	59	35 (19–464)	** < 0.0001**

Significant level was *p* < 0.05. Significant p-values are marked with bold text

#### Symptoms and treatment at follow-up

**Fig. 2:**
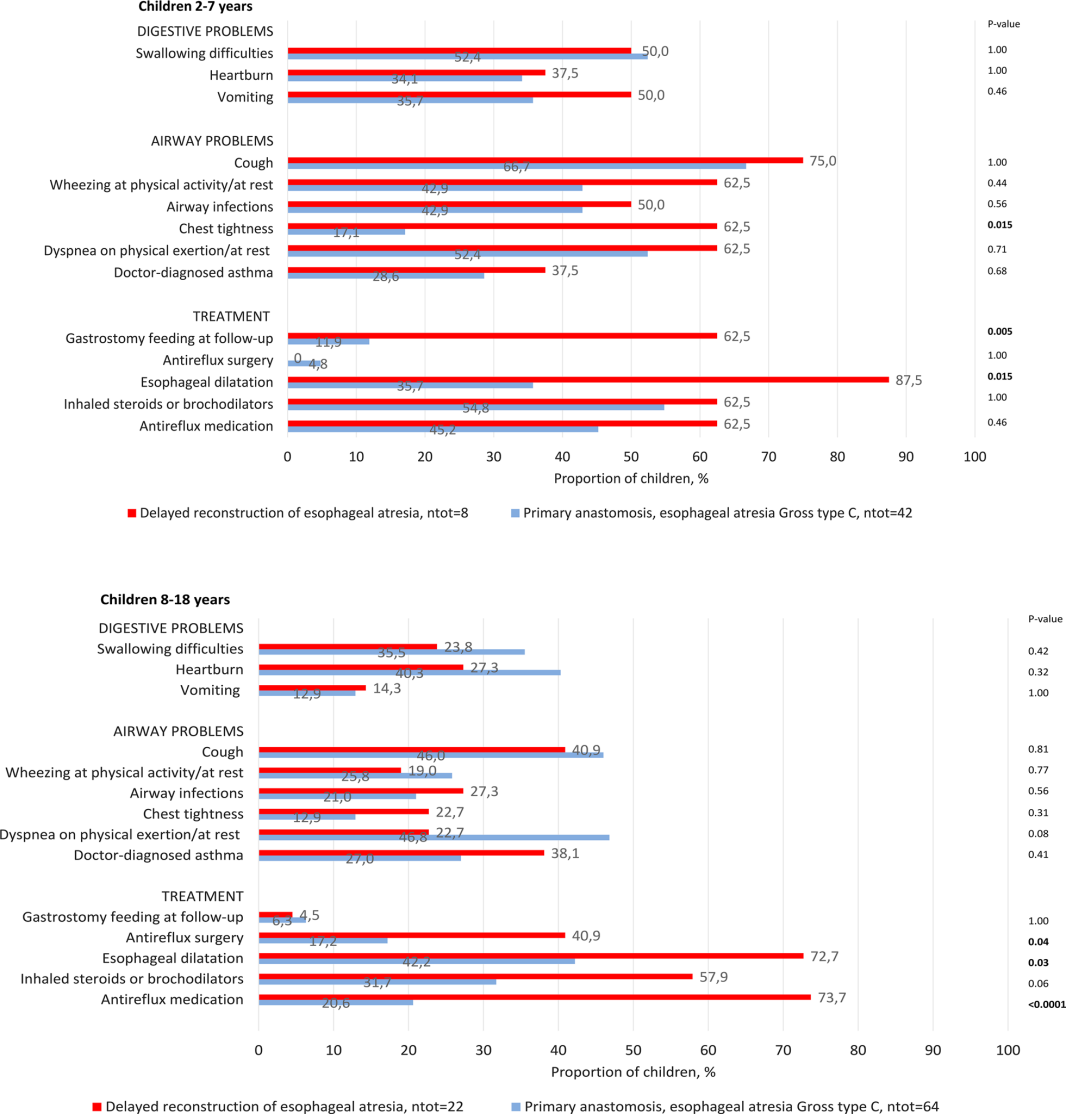
Symptom prevalence and treatments at follow up in children aged 2–7 (Fig. 2a) and in children aged 8–18 (Fig. 2b) with delayed reconstruction of esophageal atresia compared to children with esophageal atresia Gross type C who underwent primary anastomosis. The statistical comparison was performed using Fisher’s exact test. Significant level was p < 0.05. Significant p-values are marked with bold text

Figure 2a–b compares the proportion of children with DREA with digestive or respiratory symptoms and their treatment at follow-up, with that of children with PA.

In children aged 2–7 with DREA, symptom prevalence ranged from 37.5% (heartburn) to 75% (cough). There was a higher rate of chest tightness among children with DREA compared to those with PA (p = 0.015). At follow-up, more children with DREA had gastrostomy feeding (p = 0.005) and esophageal dilatations (p = 0.015), with a median of 4 (range 0–19) compared to children with PA median of 0 (range 0–11), p < 0.001. They were rarely treated with antireflux surgery, but commonly with antireflux medication, inhaled steroids and/or bronchodilators.

In children aged 8–18 with DREA, symptom prevalence ranged from 14.3% (vomiting) to 40.9% (cough). No significant differences between children with DREA vs PA regarding symptom prevalence were found. At follow-up, gastrostomy feeding was rare in any group, but children with DREA had significantly more dilatations (median 6, range 0–46) than children with PA (median 0, range 0–62), p < 0.001, as well as antireflux surgery and antireflux medication (p < 0.05). Moreover, within the DREA group, there were no significant differences in symptom prevalence between children with DPA and ER (p > 0.05). However, children with DPA were more commonly treated with esophageal dilatations (p = 0.012) than children with PA and more children with ER had antireflux surgery than children with PA (p = 0.008).

### HRQOL

**Fig. 3:**
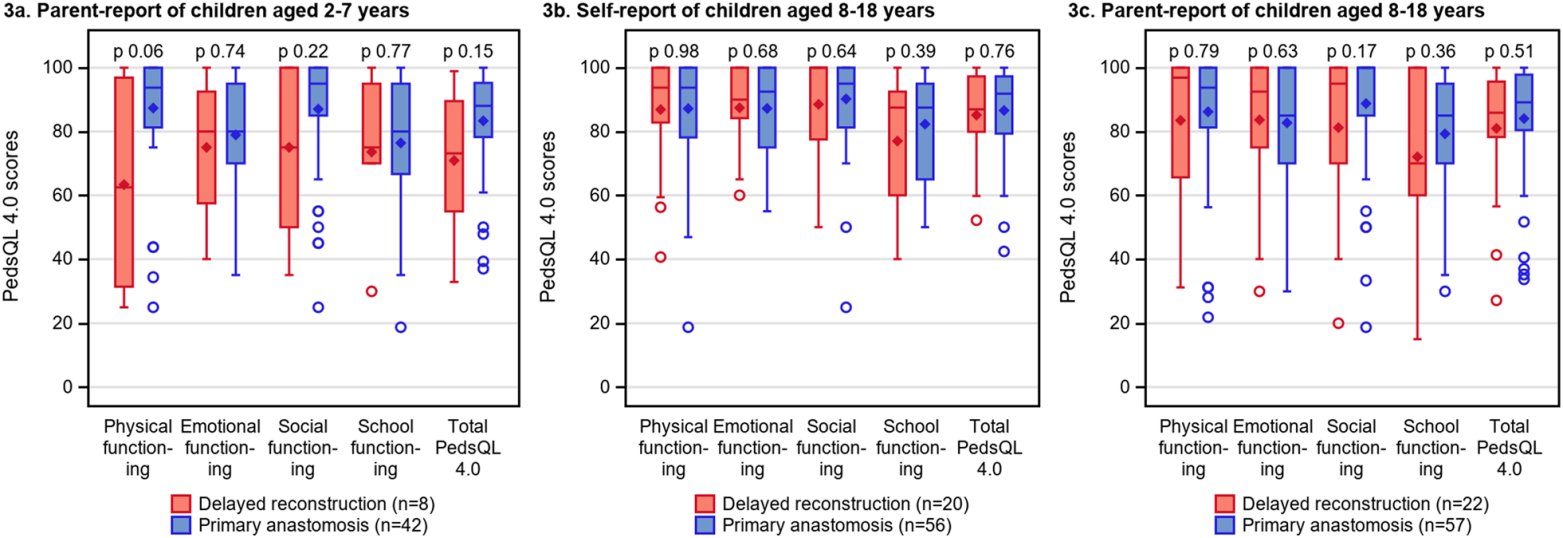
The PedsQL 4.0 scores in children aged 2–7 (**a**) and children aged 8–18 (**b**–**c**) with delayed reconstruction of esophageal atresia (including both delayed primary anastomosis and esophageal replacement) compared to children with primary anastomosis of the same age group and gender distribution

**Fig. 4:**
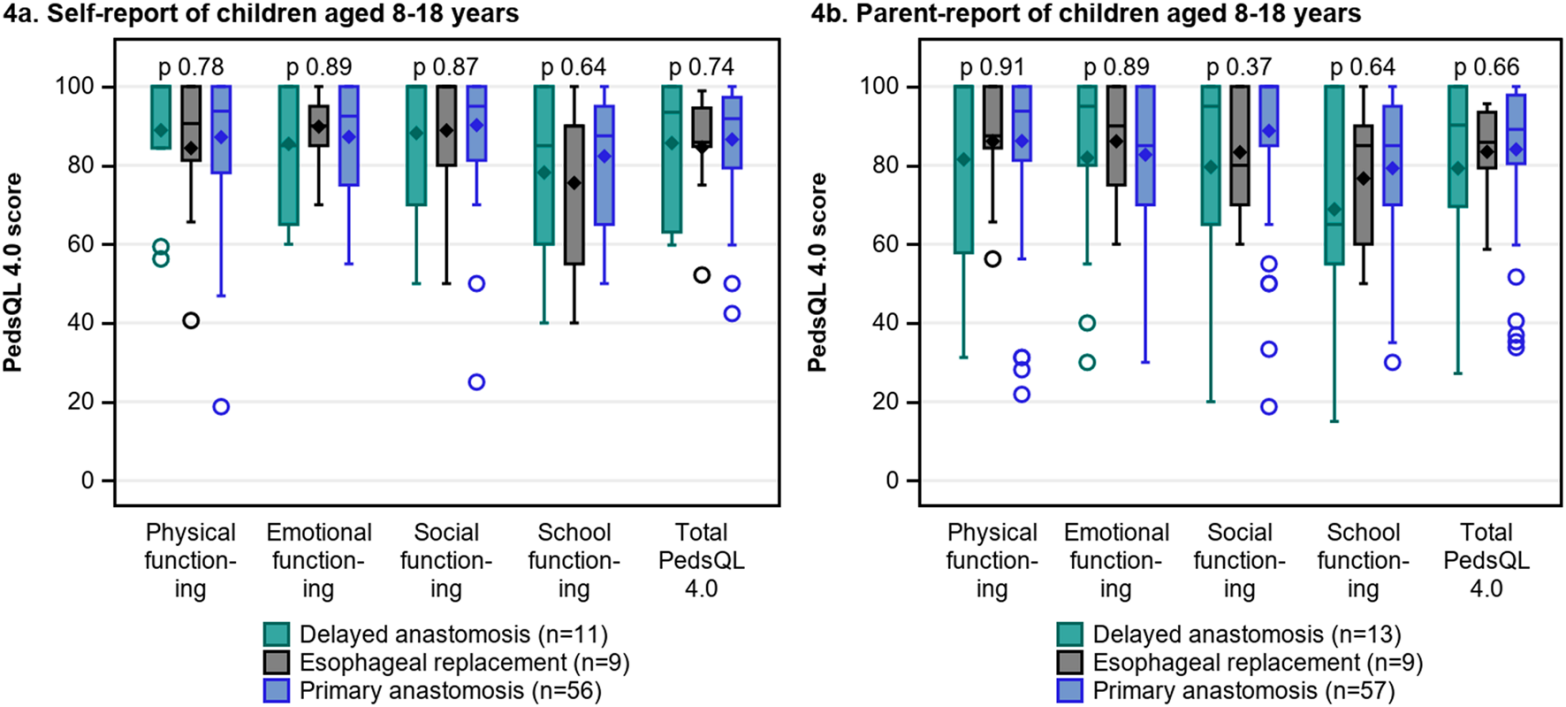
The PedsQL 4.0 scores in children aged 8–18 with delayed primary anastomosis, esophageal replacement and primary anastomosis, self-report (**a**) and parent-report (**b**)

**Fig. 5:**
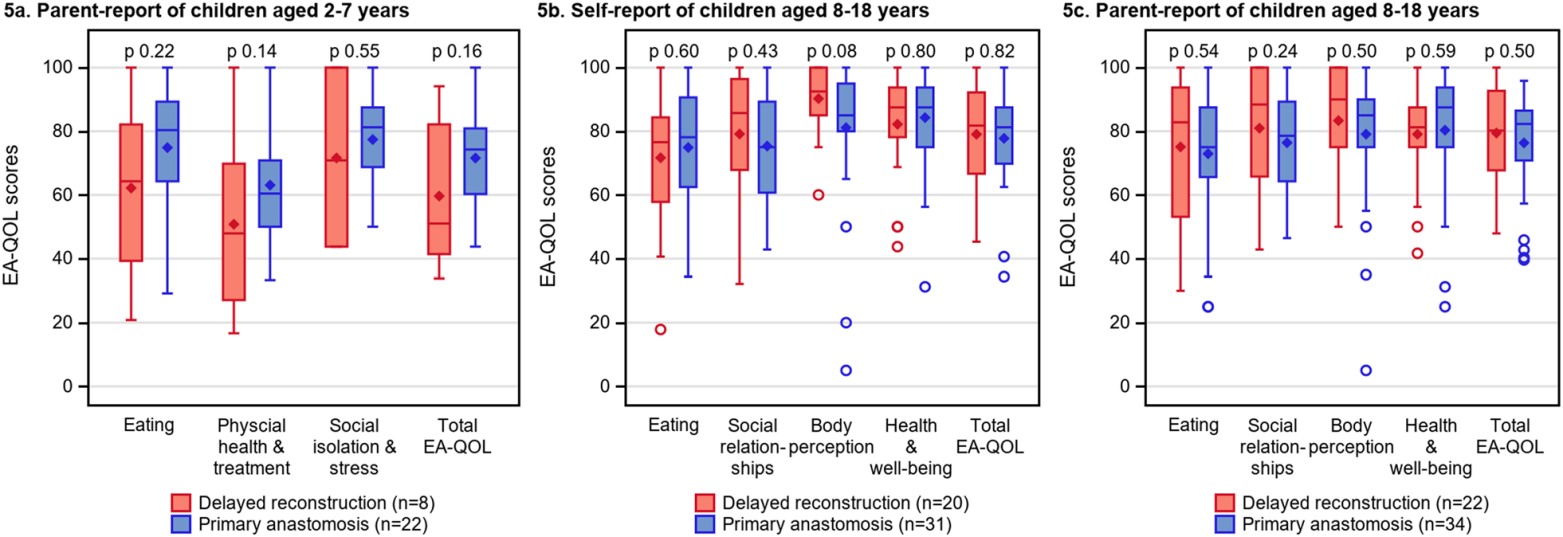
The EA-QOL scores in children aged 2–7 (**a**) and children aged 8–18 (**b**–**c**) with delayed reconstruction of esophageal atresia (including both delayed primary anastomosis and esophageal replacement) compared to children with primary anastomosis of the same age group and gender distribution

**Fig. 6:**
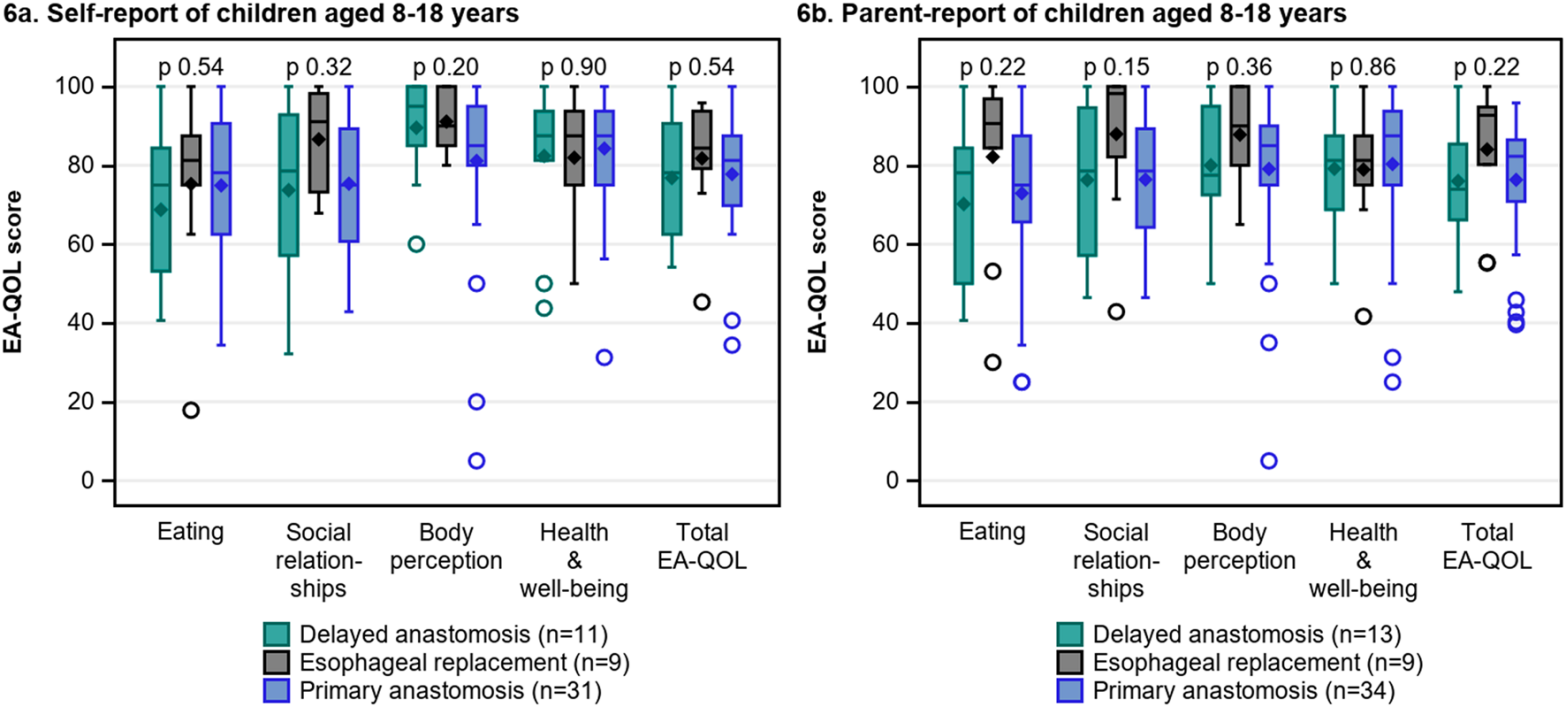
The EA-QOL scores in children aged 8–18 with delayed primary anastomosis, esophageal replacement and primary anastomosis, self-report (**a**) and parent-report (**b**)

Additional file 2 presents descriptives for generic and condition-specific HRQOL scores in children with DREA and PA, complementary to Figs. 3, 4, 5, 6.

### Generic HRQOL

Figure 3a–c compares the PedsQL 4.0 scores in children with DREA with children with PA. In children aged 2–7, the median scores for physical, social, school functioning and total generic HRQOL were numerically lower in children with DREA than with PA, but the differences were non-significant (p > 0.05). In children aged 8–18, there were no significant differences in generic HRQOL scores between children with DREA and PA (p > 0.05) or as viewed in Fig. 4a–b, between those with DPA, ER or PA (p > 0.05).

### Condition-specific HRQOL

Figure 5a–c compares the EA-QOL scores in children with DREA with children with PA. In age group 2–7, all domain or total scores measured by the EA-QOL questionnaires demonstrated lower median scores in children with DREA than in children with PA, but differences were non-significant (p > 0.05). In age group 8–18, there were no significant differences in EA-QOL scores between children with DREA and PA (p > 0.05), or as viewed in Fig. 6a–b between those with DPA, ER or PA (p > 0.05).

### Factors associated with lower HRQOL scores

Table 3 presents generic and condition-specific HRQOL in children aged 8–18 with DREA with and without digestive and respiratory symptoms (in subgroups with ≥ 5 observations). Swallowing difficulties, heartburn, cough or airway infections were significantly associated with lower generic and/or condition-specific HRQOL scores, p < 0.05.

**Table 3:** Generic and condition-specific health-related quality of life in symptom subgroups of children with delayed reconstruction of esophageal atresia

		Physical functioning					Emotional functioning					Social functioning				
Symptoms the past four weeks		n, yes	Median(range)	n, no	Median(range)	p-value	n, yes	Median(range)	n, no	Median(range)	p-value	n, yes	Median(range)	n, no	Median(range)	p-value
*Generic health-related quality of life*																
Child-Report	Swallowing difficulties	5	59.4 (40.6–100)	15	93.8 (65.6–100)	0.21	5	70.0 (60.0–100)	15	90.0 (65.0–100)	0.37	5	70.0 (50.0–100)	15	100 (50.0–100)	0.16
	Heartburn	5	87.5 (40.6–93.8)	15	100 (56.3–100)	0.054	5	85.0 (65.0–100)	15	95.0 (60.0–100)	0.35	5	95.0 (50.0–100)	15	100 (50.0–100)	0.26
	Cough	7	87.5 (40.6–100)	13	100 (65.6–100)	**0.046**	7	85.0(60.0–100)	13	95.0 (65.0–100)	0.29	7	95.0 (50.0–100)	13	100(50.0–100)	0.21
	Airway infections	5	84.4 (40.6–93.8)	15	100 (59.4–100)	**0.025**	5	70.0 (60.0–100)	15	95.0 (65.0–100)	0.075	5	70.0 (50.0–100)	15	100 (55.0–100)	**0.020**
Parent-report	Swallowing difficulties	5	56.3 (56.3–100)	17	98.4 (31.3–100)	0.091	5	80.0 (55.0–100)	17	95.0 (30.0–100)	0.35	5	67.5 (60.0–100)	17	95.0 (20.0–100)	0.36
	Heartburn	6	57.8 (31.3–87.5)	16	100 (56.3–100)	**0.003**	6	70.0 (30.0–100)	16	95.0 (55.0–100)	**0.041**	6	67.5 (20.0–100)	15	95.0 (60.0–100)	0.084
	Cough	8	57.8 (31.3–87.5)	13	100 (65.6–100)	** < 0,001**	9	80.0 (30.0–100)	13	95.0 (70.0–100)	**0.026**	8	67.5 (20.0100)	13	100 (70.0–100)	**0.036**
	Airway infections	6	57.8 (31.2–100)	15	100 (56.3–100)	**0.017**	6	57.5 (30.0–100)	16	95.0 (70.0–100)	0.096	6	60.0 (20.0–100)	15	95.0 (70.0–100)	**0.009**

		School functioning					Total PedsQL 4.0 scores				
Symptoms the past four weeks		n, yes	Median(range)	n, no	Median(range)	p-value	n, yes	Median(range)	n, no	Median(range)	p-value
*Generic health-related quality of life*											
Child-Report	Swallowing difficulties	5	60.0 (55.0–100)	11	90.0 (40.0–100)	0.40	5	60.9 (52.2–100)	15	87.0 (63.0–100)	0.19
	Heartburn	5	60.0 (55.0–65.0)	15	90.0 (40.0–100)	**0.027**	5	84.8 (52.2–87.0)	15	94.6 (61.0–100)	**0.044**
	Cough	7	60.0 (55.0–100)	13	94.6 (63.0–100)	0.077	7	84.8 (52.2–100)	13	93.5 (78.3–100)	0.080
	Airway infections	5	55.0 (40.0–65.0)	15	90.0 (40.0–100)	**0.006**	5	63.0 (52.2–87.0)	15	94.6 (60.0–100)	**0.014**
Parent-report	Swallowing difficulties	5	60.0 (55.0–100)	17	55.0 (15.0–100)	0.61	5	69.6 (56.5–100)	17	85.9 (27.2–100)	0.19
	Heartburn	6	55.0 (15.0–75.0)	16	87.5 (35.0–100)	**0.009**	6	64.1 (27.2–85.9)	16	91.8 (56.5–100)	**0.009**
	Cough	9	60.0 15.0–100)	13	90.0 (35.0–100)	**0.017**	9	69.6 (27.2–100)	13	93.5 (78.3–100)	**0.007**
	Airway infections	6	45.0 (15.0–60.0)	16	87.5 (50.0–100)	**0.001**	6	57.6 (27.2–85.9)	16	91.8 (69.6–100)	**0.004**

		Eating					Social relationships					Body perception				
Symptoms the past four weeks		n, yes	Median(range)	n, no	Median(range)	p-value	n, yes	Median(range)	n, no	Median(range)	p-value	n, yes	Median(range)	n, no	Median(range)	p-value
*Condition-specific health-related quality of life*																
Child report	Swallowing difficulties	5	43.8 (17.9–59.4)	14	81.3 (53.1–100)	**0.002**	5	66.1 (57.1–85.7)	14	91.1 (42.9–100)	**0.048**	5	90.0 (75.0–95.0)	14	95.0 (80.0–100)	0.13
	Heartburn	5	75.0 (17.9–84.4)	15	78.1 (43.8–100)	0.29	5	73.2 (57.1–89.3)	15	89.3 (32.1–100)	0.27	5	85.0 (75.0–100)	15	95.0 (60.0–100)	0.30
	Cough	7	59.4 (17.9–84.4)	13	81.3 (43.8–100)	0.080	6	73.2 (57.1–89.3)	13	92.8 (32.1–100)	0.13	7	95.0 (75.0–100)	13	90.0 (60.0–100)	0.54
	Airway infections	5	59.4 (17.9–84.4)	15	78.1 (40.6–100)	0.26	5	66.1 (42.9–89.3)	15	89.3 (32.1–100)	0.11	5	95.0 (80.0–100)	15	90.0 (60.0–100)	0.86
Parent-report	Swallowing difficulties	5	50.0 (30.0–60.3)	16	87.5 (50.0–100)	**0.002**	5	74.1 (53.6–89.3)	16	96.4 (42.9–100)	0.15	5	80.0 (50.0–95.0)	16	90.0 (65.0–100)	0.51
	Heartburn	6	51.6 (30.0–96.9)	16	84.4 (50.0–100)	**0.024**	5	62.2 (42.9–71.4)	16	94.6 (46.4–100)	**0.020**	5	65.0 (50.0–80.0)	16	92.5 (55.0–100)	**0.006**
	Cough	9	53.1 (30.0–96.9)	13	90.6 (50.0–100)	**0.004**	9	71.4 (42.9–89.3)	13	100 (46.4–100)	**0.039**	9	72.5 (50.0–95.0)	13	90.0 (55.0–100)	**0.030**
	Airway infections	6	56.3 (30.0–96.9)	16	84.4 (40.6–100)	0.10	5	71.1 (46.4–89.3)	16	94.6 (42.9–100)	0.12	5	75.0 (65.0–95.0)	16	90.0 (50.0–100)	0.19

		Health & well-being					Total EA-QOL				
Symptoms the past four weeks		n, yes	Median(range)	n, no	Median(range)	p-value	n, yes	Median(range)	n, no	Median(range)	p-value
*Condition-specific health-related quality of life*											
Child report	Swallowing difficulties	5	50.0 (43.8–87.5)	14	90.6 (68.8–100)	**0.031**	5	64.6 (45.3–78.1)	14	88.5 (62.5–100)	**0.0046**
	Heartburn	5	68.8 (50.0–93.8)	15	87,5 (43.8–100)	0.13	5	79.2 (45.3–90.6)	15	85.4 (61.5–100)	0.15
	Cough	7	68.8 (43.8–93.8)	13	87.5 (75.0–100)	**0.037**	7	78.1 (45.3–90.6)	13	86.5 (61.5–100)	0.052
	Airway infections	5	69.8 (43.8–93.8)	15	87.5 (50.0–100)	**0.046**	5	64.6 (45.3–90.6)	15	85.4 (54.2–100)	0.097
Parent-report	Swallowing difficulties	5	56.3 (41.7–81.3)	16	87.5 (62.5–100)	**0.011**	5	64.6 (47.9–74.0)	16	90.6 (55.2–100)	**0.013**
	Heartburn	5	62.5 (41.7–81.3)	16	87.5 (56.3–100)	**0.007**	5	55.4 (47.9–80.1)	16	90.1 (64.6–100)	**0.006**
	Cough	9	65.6 (41.7–81.3)	13	87.5 (75.0–100)	**0.001**	9	69.0 (47.9–80.2)	13	92.7 (64.6–100)	**0.006**
	Airway infections	5	62.5 (41.7–87.5)	16	84.4 (50.0–100)	0.072	5	69.8 (55.4–80.2)	16	90.1 (47.9–100)	0.090

Significant level was *p* < 0.05. Significant *p*-values are marked with bold text

Table 4 shows the correlation between clinical factors and HRQOL scores among children with DREA. The number of associated anomalies present in the child demonstrated a strong negative correlation with six HRQOL scales in children aged 2–7, p < 0.05. Similarly, days to discharge from tertiary pediatric surgical ward showed a strong negative correlation with five HRQOL scales in children aged 2–7, p < 0.05.

**Table 4:** Correlation between HRQOL scores and clinical factors among children with delayed reconstruction of EA

	Spearman’s rho					
Generic health− related quality of life	Gestational weeks at birth	Birth weight grams	Number of associated anomalies^a^	Days to reconstruction	Days to discharge from tertiary pediatric surgical ward	Number of esophageal dilatations
*Children aged 2–7 years (parent− reports, n = 8)*						
Physical functioning	− 0.38	− 0.61	− 0.83*	− 0.68	− 0.76*	0.074
Emotional functioning	− 0.61	− 0.74*	− 0.80*	− 0.62	− 0.68	0.11
Social functioning	− 0.15	− 0.27	− 0.72	− 0.52	− 0.78*	0.12
School functioning	− 0.15	− 0.31	− 0.72	− 0.46	− 0.71	0.055
Total PedsQL 4.0 scores	− 0.34	− 0.60	− 0.80*	− 0.62	− 0.79*	− 0.024
*Children aged 8–18 years (child− reports, n = 20/parent− reports, n = 22)*						
Physical functioning	0.04/− 0.06	0.13/− 0.03	− 0.40/− 0.36	0.18/0.23	− 0.043/ − 0.007	0.042/0.37
Emotional functioning	− 0.12/0.15	0.20 /0.29	− 0.052/− 0.25	0.14/− 0.46	0.063/− 0.16	0.053/0.33
Social functioning	0.04/0.023	0.23/0.28	− 0.15/− 0.05	0.21/0.04	− 0.02/0.041	− 0.12/0.14
School functioning	− 0.04/− 0.003	0.008/0.05	− 0.25/− 0.29	0.051/− 0.08	− 0.15/− 0.11	0.11/0.27
Total PedsQL 4.0 scores	− 0.07/0.04	0.03 /0.11	− 0.37/− 0.37	0.14/0.06	− 0.14/− 0.12	0.082/0.26
*Condition− specific health− related quality of lifE*						
*Children aged 2–7 years (parent− reports, n = 8)*						
Eating	− 0.67	− 0.72	− 0.81*	− 0.64	− 0.71	− 0.074
Physical health & treatment	− 0.39	− 0.62	− 0.80*	− 0.68	− 0.74*	0.024
Social isolation & stress	0.38	0.10	− 0.40	− 0.30	− 0.65	− 0.077
Total EA− QOL	− 0.34	− 0.54	− 0.78*	− 0.57	− 0.83*	− 0.073
*Children aged 8–18 years (child− reports, n = 20/parent− reports, n = 22)*						
Eating	0.10/0.10	0.23/0.17	− 0.10/− 0.12	− 0.018/− 0.12	− 0.17/− 0.24	− 0.001/0.10
Social relationships	0.10/0.13	0.12/0.028	− 0.02/0.28	0.19/0.11	− 0.10/− 0.04	0/0.02
Body perception	0.21/0.15	0.25/0.037	− 0.1/0.24	0.07/0.35	− 0.18/0.08	− 0.096/0.03
Health & well− being	0.10/− 0.01	0.02 /− 0.03	− 0.53*/− 0.35	− 0.08/− 0.08	− 0.37/− 0.28	0.21/0.37
Total EA− QOL	0.13/0.07	0.24/0.09	− 0.14/0.003	0.13/0.04	− 0.12/− 0.14	0.07/0.17

*HRQOL* Health-related quality of life, *EA*  Esophageal atresia

*p < 0.05

Spearman’s rho considered weak (0–0.39), moderate (0.40–0.59), strong (≥ 0.60)

^a^Cardio-vascular, gastrointestinal, urogenital, limb, vertebrae-rib, choanalatresia, eye, ear, central nervous system or respiratory anomaly or other

### Child-parent agreement regarding the child’s HRQOL

Table 5 presents the parent–child agreement in ratings of the child’s generic and condition-specific HRQOL in children aged 8–18 with DREA, with the ICCs indicating moderate to good parent–child agreement.

**Table 5:** Parent–child agreement in ratings of the child’s generic and condition-specific HRQOL in children aged 8–18 years with delayed reconstruction of esophageal atresia

	Intraclass correlation coefficients average measures
*Generic health-related quality of life (PedsQL 4.0)*	
Physical functioning	0.80
Emotional functioning	0.68
Social functioning	0.61
School functioning	0.87
Total PedsQL 4.0 scores	0.76
*Conditions-specific health-related quality of life (EA-QOL)*	
Eating	0.88
Social relationships	0.81
Body perception	0.74
Health & well-being	0.88
Total EA-QOL scores	0.84

Intraclass correlation coefficients considered moderate (0.5–0.74), good (0.75– 0.9) and excellent (> 0.90)

## Discussion

This is the first study to report postoperative morbidity and generic as well as condition-specific HRQOL of life in children with DREA, using a national wide recruitment and comparing outcomes to children with PA of the same age group and gender distribution. Overall, we found that children with DREA do not present with more long-term digestive and respiratory morbidity or impaired HRQOL than children with PA.

Among our participants, the reconstruction was delayed mostly because of LGEA, which commonly refers to a gap length of ≥ 2–3 cm or ≥ 3 vertebral bodies [3, 8] and although it is debated, LGEA can entail Gross type A, B and C [4], which is confirmed in our study. In line with previous literature on LGEA [18, 19], DREA was related to a higher frequency of associated anomalies in children aged 2–7 as well as to prematurity and low birth weight in children aged 8–18. However, in contrast [18, 19], genetic disorders were similarly present in children with DREA and PA. In our study sample, 25% of children aged 2–7 and 54% of those aged 8–18 had DPA, which has been advocated as the best choice in LGEA [46, 47]. Evidence for one conduit being superior to another is weak [3, 4]. Altogether, gastric tube was most used, but the Swedish sample showed variety regarding ER. Currently, GPU is favored by several institutions, probably due to its technical safety [8] and has been introduced on Swedish children aged 2–7.

We observed that early postoperative complications were generally more common in children with DREA, but differences with children with PA were mostly non-significant. In terms of late morbidity, cough was the most reported symptom in children with DREA, possibly due to the relationship with tracheomalacia, GERD, esophageal strictures, airway infections and asthma [48]. The underlying pulmonary morbidity affecting all the subgroups may be also related to a disturbed development and maturation of the respiratory tract seen in laboratory animals and in clinical patients with EA [49]. In children aged 2–7 with DREA, the least reported symptom was heartburn and antireflux medication was commonly used. Antireflux surgery however, was rarely employed in children aged 2–7, which is in line with recent studies suggesting restrictiveness [50]. Furthermore, none of the four children who were reported vomiting showed esophagitis according to biopsies. In children aged 8–18 with DREA, the least reported symptom was vomiting, but in this group the use of antireflux treatment may serve as explanation. A majority of children with DREA were treated with dilatations at follow-up. In age group 8–18, esophageal dilatations were most common after DPA, which is in agreement with findings by Stadil et al. [51]. In follow-ups of children with DREA several children aged 2–7 were still dependent on gastrostomy feeding, unlike children aged 8–18.

Interestingly, when comparing the presence of respiratory or digestive symptoms in children with DREA and PA, there were very few significant differences. In children aged 2–7, these symptoms and medical treatments were common in both groups [23, 29, 52], which may explain these findings. Though, chest tightness was more frequent in children with DREA. The use of GPU has previously been associated with chest tightness [53], but while only three children had GPU in our sample, the sample size is too small to find definite explanations In children aged 8–18, no significant differences in symptom prevalence between children with DREA and PA were seen, despite a high frequency of GER(D) and strictures being reported in patients with DPA, gastric tube and partial GPU [53, 54]. Since 2011, children with EA in Sweden are offered a standardized follow-up according to a pediatric surgical programme, and more care when needed. Children with PA were recruited from a center which has applied a standardized follow-up programme since the late 1990s. Nevertheless, more children aged 8–18 with DREA than with PA were treated for digestive morbidity and 58% used inhaled steroids and/or bronchodilators. This could imply that children with DREA, a group where complications are expected, have received more intense follow-up/treatments. In turn, this may explain their comparable symptom prevalence to children with PA.

Our study findings comparing HRQOL in children with DREA and PA agree with most previous studies showing similar levels between patients with LGEA/complicated EA and those with PA or with healthy references [33, 35, 36, 55]. Although these studies differ in design, HRQOL assessments and subgroups of children with LGEA/complex EA, they focus on a complicated group of patients with EA. As previously discussed [35, 55–58], the HRQOL results may be explained by the congenital nature of EA, where disease-related challenges become a part of the children’s identity [59] and adaptation [60, 61]. There are only two studies of coping used by children with EA [60, 61], and these demonstrate that already as toddlers they use coping strategies in several disease-specific contexts. Their use of coping strategies is related to the severity of EA and can impact the children’s HRQOL both positively and negatively [60]. Hence, there should be more research into coping as a possible factor influencing HRQOL in children with EA.

In children aged 8–18 with DREA, the presence of digestive or respiratory symptoms were associated with worse generic and/or condition-specific HRQOL, as in children with EA in general [42, 56, 62]. Like Gallo et al. [35], we could not confirm a relationship between esophageal dilatations and HRQOL. However, esophageal dilatations may reflect disease severity, treatment aims to relieve troublesome symptoms [63]. Moreover, prematurity and low birth weight were not associated with impaired HRQOL in children with DREA. This differs to findings in studies including complicated/complex and mild cases of EA [34, 39], where these variables could be interlinked with LGEA and associated anomalies. To the authors’ knowledge, we are the first to show that an initial long hospital stay on a tertiary pediatric surgical ward, and a number of associated anomalies, acting as possible markers of disease severity negatively influenced HRQOL in children aged 2–7 with DREA. Moreover that in the DREA group, child/parent agreement as to the child’s HRQOL was acceptable, in line with studies including children with mild and complicated EA [64].

### Limitations

As in other studies [3, 4, 14], surgical treatments of DREA in Sweden vary according to institution and surgeon. Techniques like Foker [11, 12] or Kimura [13], jejunal interposition or thoracoscopic repair have not yet been introduced, which may differ to other countries. Despite nation-wide recruitment, the study sample is small, but larger than several HRQOL studies, including ≤ 10 children in subgroups of complex EA [35, 36, 55]. Study sample inclusion was 30/45(67%), the overall response rate 30/34(88%) and respondents and non-respondents had similar Gross type and surgical procedures. Still, the number of non-participants weakens the study’s generalizability. The group of children is heterogenous in relation to indications for DREA, anatomical subtype, gap measurement, prematurity and associated anomalies. However, if we had applied more exclusion criteria to increase sample homogeneity, the study size a nationalwide Swedish study would have been limited. The study did not use a control group of healthy children. Although we paused data collection until the implications of the covid-19 pandemic were better understood, the situation could hypothetically impact the HRQOL results [40, 65].

## Conclusions

In a nation-wide Swedish setting, children with DREA do not overall present with more long-term postoperative morbidity or lower generic and condition-specific HRQOL than children with PA of the same age group and gender distribution. This supports an understanding that children with DREA are not necessarily a risk group for impaired HRQOL compared with children with PA. However, in children with DREA, risk factors for impaired HRQOL may be an initial long hospital stay, several associated anomalies and persistent airway and digestive symptoms. Moreover, parents can probably be a reliable source of information, complementary to self-reporting in ages 8–18. This is important and encouraging information for clinical practice, parents, children and patient stakeholders. Nevertheless, there is a need for an international multicenter study focusing on HRQOL, coping/adaption and health care experiences in treatment groups of children with LGEA/complex EA and PA.

## Supplementary Information

**Additional file 1.** Details the congenital/neonatal characteristics of children with DPA, ER and PA aged 8–18, characteristics of their parent-proxy, their postoperative morbidity and treatment at follow-up.

**Additional file 2.** Presents descriptives for generic and condition-specific HRQOL scores in children with DREA and PA, complementary to Figs. 3, 4, 5, 6.

## Data Availability

The datasets analyzed during the current study are available in the manuscript or in its additional files. Further information is not available in public due to lack of ethical approval.

## Abbreviations

DREA: Delayed reconstruction of esophageal atresia
DPA: Delayed primary anastomosis
EA: Esophageal atresia
EA-QOL: Esophageal-Atresia-quality-of life
ER: Esophageal replacement
GERD: Gastroesophageal reflux disease
GPU: Gastric pull-up
HRQOL: Health-related quality of life
LGEA: Long-gap esophageal atresia
PA: Primary anastomosis
PedsQL 4.0^™^: Pediatric quality of life inventory
TEF: Tracheo-esophageal fistula
