# Supplementary material for: Postoperative morbidity and health-related quality of life in children with delayed reconstruction of esophageal atresia: a nationwide Swedish study
Source: Orphanet J Rare Dis. 2022 Jun 20;17:239. doi: 10.1186/s13023-022-02381-y (PMC9207832; doi:10.1186/s13023-022-02381-y)
Supplement: Supplementary file 1 — Additional file 1. Details the congenital/neonatal characteristics of children with DPA, ER and PA aged 8–18, characteristics of their parent-proxy, their postoperative morbidity and treatment at follow-up. [file 13023_2022_2381_MOESM1_ESM.docx]

# Additional file 1.

Characteristics of children with esophageal atresia and delayed primary anastomosis, esophageal replacement and primary anastomosis were compared using Pearson Chi Square test, i.e to investigate if there were any significant differences between the three groups. If so, a post hoc Fisher's exact test was employed to determine between which two groups there was a significant difference. Significant level p<0.05.

| Congenital and postoperative characteristics in surgical subgroups of children with esophageal atresia aged 8-18 years | | | | | | | |
| --- | --- | --- | --- | --- | --- | --- | --- |
|  | **DPA** | | **ER** | |  | **PA** |  |
| **Child congenital characteristics** | **n_tot_** | **n(%)** | **n_tot_** | **n(%)** | **n_tot_** | **n(%)** | **p-value, Pearson Chi Square test** |
| Child gender male | 12 | 5(41.7) | 10 | 5(50.0) | 64 | 34(53.1) | 0.76 |
| Prematurely born (< 37 gestational weeks) | 12 | 7(58.3) | 10 | 6(60.0) | 62 | 17(27.4) | **0.029** |
| Low birth weight (< 2500 grams) | 12 | 7(58.3) | 10 | 6(60.0) | 61 | 18(29.5) | **0.048** |
| Associated anomalies^a^ | 12 | 5(41.7) | 10 | 7(70.0) | 64 | 39(60.9) | 0.35 |
| Cardiovascular | 12 | 3(25.0) | 10 | 2(20.0) | 64 | 22(34.4) | 0.60 |
| Anorectal | 12 | 1(8.3) | 10 | 4(40.0) | 64 | 4(6.3) | **0.005** |
| Urogenital | 12 | 2(16.7) | 10 | 5(50.0) | 64 | 8(12.5) | **0.015** |
| VACTERL assocation^b^ | 12 | 0 | 10 | 3(30.0) | 64 | 11(17.2) | 0.15 |
| Verified genetic disorder | 12 | 1(8.3) | 10 | 3(30.0) | 64 | 7(10.9) | 0.14 |
|  |  |  |  |  |  |  |  |
| **Early postoperative course before discharge from tertiary pediatric surgical ward** |  |  |  |  |  |  |  |
| Anastomotic leakage | 12 | 3(25.0) | 10 | 5(50.0) | 62 | 8(12.9) | **0.018** |
| Revisional surgery after repair of esophageal atresia | 12 | 3(25.0) | 10 | 1(10.0) | 64 | 7(10.9) | 0.40 |
| Sepsis verified through blood culture | 12 | 2(16.7) | 10 | 4(40.0) | 62 | 6(9.7) | **0.038** |
| Wound infection | 12 | 0 | 10 | 1(10.0) | 62 | 2(3.2) | 0.44 |
| Pneumothorax treated with drainage | 12 | 3(25.0) | 10 | 0 | 62 | 11(17.7) | 0.27 |
| Esophageal dilatation before hospital discharge | 11 | 0 | 9 | 2(22.2) | 60 | 8(13.3) | 0.30 |
| Days to discharge from tertiary pediatric surgical care, median (range) | 12 | 163(63-364) | 10 | 206(62-347) | 59 | 35(19-364) | **<0.0001** |
| DPA=delayed primary anastomosis, ER= esophageal replacement, PA=primary anastomosis  ^a^ cardio-vascular, gastrointestinal, urogenital, limb, vertebrae-rib, choanalatresia, eye, ear, central nervous system or respiratory anomaly  *^b^* stands for vertebral defects, anal atresia, cardiac defects, tracheo-esophageal fistula, renal anomalies, and limb abnormalities. People diagnosed with VACTERL association typically have at least three of these characteristic features  Congenital characteristics and postoperative course before discharge from tertiary pediatric surgical ward in children aged 8-18 years who had delayed reconstruction of esophageal atresia with DPA or ER compared to children with esophageal atresia and distal tracheoesophageal fistula who had PA | | | | | | | |

| Symptoms and treatment in surgical subgroups of children with esophageal atresia aged 8-18 years | | | | | | | |
| --- | --- | --- | --- | --- | --- | --- | --- |
|  | **DPA** | | **ER** | | **PA** | |  |
|  | **n_tot_** | **n(%)** |  | **n(%)** | **n_tot_** | **n(%)** | **p-value,** **Pearson Chi Square test** |
| **Digestive problems** |  |  |  |  |  |  |  |
| Swallowing difficulties | 11 | 4(36.4) | 10 | 1(10.0) | 62 | 22(35.5) | 0.27 |
| Heartburn | 12 | 3(25.0) | 10 | 3(30.0) | 62 | 25(40.3) | 0.54 |
| Vomiting | 11 | 2(18.2) | 10 | 1(10.0) | 62 | 8(12.9) | 0.88 |
|  |  |  |  |  |  |  |  |
| **Airway problems** |  |  |  |  |  |  |  |
| Cough | 12 | 6(50.0) | 10 | 3(30.0) | 63 | 29(46.0) | 0.59 |
| Wheezing | 11 | 3(27.3) | 10 | 1(10.0) | 62 | 16(25.8) | 0.54 |
| Airway infections | 12 | 4(33.3) | 10 | 2(20.0) | 62 | 13(21.0) | 0.63 |
| Dyspnea | 12 | 3(25.0) | 10 | 2(20.0) | 62 | 29(46.8) | 0.14 |
| Chest tightness | 12 | 3(25.0) | 10 | 2(20.0) | 62 | 8(12.9) | 0.52 |
| Doctor-diagnosed asthma | 11 | 4(36.4) | 10 | 4(40.0) | 63 | 17(27.0) | 0.62 |
|  |  |  |  |  |  |  |  |
| **Treatment** |  |  |  |  |  |  |  |
| Antireflux surgery | 12 | 3(25.0) | 10 | 6(60.0) | 64 | 11(17.2) | **0.012** |
| Dilatation | 12 | 10(83.3) | 10 | 6(60.0) | 64 | 27(42.2) | **0.026** |
| Antireflux medication | 9 | 6(66.7) | 10 | 8(80.0) | 63 | 13(20.6) | **<0.0001** |
| Inhaled sterioids or bronchodilators | 9 | 6(66.7) | 10 | 5(50.0) | 63 | 20(31.7) | 0.091 |
| DPA=delayed primary anastomosis, ER= esophageal replacement, PA=primary anastomosis  Symptom and treatment at follow-up in children aged 8-18 years who had delayed reconstruction of esophageal atresia and underwent DPA or ER compared to children with esophageal atresia and distal tracheoesophageal fistula who had PA | | | | | | | |
