# Supplementary material for: Postoperative morbidity and health-related quality of life in children with delayed reconstruction of esophageal atresia: a nationwide Swedish study
Source: Orphanet J Rare Dis. 2022 Jun 20;17:239. doi: 10.1186/s13023-022-02381-y (PMC9207832; doi:10.1186/s13023-022-02381-y)
Supplement: Supplementary file 2 — Additional file 2. Presents descriptives for generic and condition-specific HRQOL scores in children with DREA and PA, complementary to Figs. 3, 4, 5, 6. [file 13023_2022_2381_MOESM2_ESM.rtf]

Additional file 2. 
Information complementary to:  
·	Figure 3a-c (Box plots) which displays the PedsQL 4.0 scores in children with delayed reconstruction of esophageal atresia (EA) aged 2-7 (Figure a) and children with delayed reconstruction of EA aged 8-18 (Figure b child-report, Figure c parent-report) compared to EA children with primary anastomosis of the same age group. Furthermore, detailed data for Figure 4a-b (Box plots) are also shown, ie regarding the PedsQL 4.0 scores of children with EA aged 8-18 who initially had delayed primary anastomosis, esophageal replacement, or primary anastomosis, pp 2-4.

·	Figure 5a-c (Box plots) which displays the EA-QOL scores in children aged 2-7 (Figure a) and aged 8-18 (Figure b child-report, Figure c parent-report) with delayed reconstruction of EA compared to EA children with PA of the same age group. Lastly, detailed data for Figure 6a-b (Box plots) are shown, ie regarding EA-QOL scores in children with EA aged 8-18 years who had primary anastomosis, esophageal replacement, or primary anastomosis, pp 5-7.


Generic health-related quality of life scores in children with delayed reconstruction of esophageal atresia (Gross A, B, C) compared to those with primary anastomosis (Gross type C)	
	scales	Mean	Std Dev	Minimum	Lower Quartile	Median	Upper Quartile	Maximum	
Children aged 2- 7 years, parent-report									
Delayed reconstruction of esophageal atresia (n=8)	Physical functioning	63.3	32.0	25.0	31.5	62.5	96.9	100.0	
	Emotional functioning	75.0	21.5	40.0	57.5	80.0	92.5	100.0	
	Social functioning	75.0	25.5	35.0	50.0	75.0	100.0	100.0	
	School functioning	73.6	22.7	30.0	70.0	75.0	95.0	100.0	
	Total PedsQL 4.0	70.9	23.0	33.0	55.0	73.1	89.5	98.9	
Primary anastomosis, esophageal atresia Gross type C (n=42)	Physical functioning	87.4	18.7	25.0	81.3	93.8	100.0	100.0	
	Emotional functioning	78.9	16.9	35.0	70.0	80.0	95.0	100.0	
	Social functioning	87.0	19.3	25.0	85.0	95.0	100.0	100.0	
	School functioning	76.4	21.4	18.8	66.7	80.0	95.0	100.0	
	Total PedsQL 4.0	83.3	16.4	37.0	78.3	88.0	95.2	100.0	
Children aged 8-18 years, child-report									
Delayed reconstruction of esophageal atresia (n=20)	Physical functioning	86.9	17.8	40.6	82.8	93.8	100.0	100.0	
	Emotional functioning	87.4	13.1	60.0	84.2	90.0	100.0	100.0	
	Social functioning	88.5	18.3	50.0	77.5	100.0	100.0	100.0	
	School functioning	77.0	20.5	40.0	60.0	87.5	92.5	100.0	
	Total PedsQL 4.0	85.2	15.1	52.2	79.9	87.0	97.3	100.0	
Primary anastomosis, esophageal atresia Gross type C (n=56)	Physical functioning	87.2	17.5	18.8	78.1	93.8	100.0	100.0	
	Emotional functioning	87.2	14.9	55.0	75.0	92.5	100.0	100.0	
	Social functioning	90.2	14.0	25.0	81.3	95.0	100.0	100.0	
	School functioning	82.3	16.7	50.0	65.0	87.5	95.0	100.0	
	Total PedsQL 4.0	86.6	14.0	42.4	79.3	91.8	97.3	100.0	
Children aged 8-18 years, parent-report									
Delayed reconstruction of esophageal atresia (n=22)	Physical functioning	83.5	21.0	31.3	65.6	96.9	100.0	100.0	
	Emotional functioning	83.6	20.8	30.0	75.0	92.5	100.0	100.0	
	Social functioning	81.2	22.6	20.0	70.0	95.0	100.0	100.0	
	School functioning	72.0	26.2	15.0	60.0	70.0	100.0	100.0	
	Total PedsQL 4.0	81.0	19.8	27.2	78.3	85.9	95.7	100.0	
Primary anastomosis, esophageal atresia Gross type C (n=57)	Physical functioning	86.2	19.8	21.9	81.3	93.8	100.0	100.0	
	Emotional functioning	82.7	17.7	30.0	70.0	85.0	100.0	100.0	
	Social functioning	88.8	18.1	18.8	85.0	100.0	100.0	100.0	
	School functioning	79.3	19.7	30.0	70.0	85.0	95.0	100.0	
	Total PedsQL 4.0	84.1	17.5	33.8	80.4	89.1	97.8	100.0	


Generic health-related quality of life scores in children aged 8-18 with delayed primary anastomosis, esophageal replacement and those with primary anastomosis (Gross type C)	
 	 	Mean	Std Dev	Minimum	Lower Quartile	Median	Upper Quartile	Maximum	
Children aged 8-18 years, child-report	Scales								
Delayed primary anastomosis (n=11)
 
 
 
 	Physical functioning	88.9	16.6	56.3	84.4	100.0	100.0	100.0	
	Emotional functioning	85.5	15.6	60.0	65.0	85.0	100.0	100.0	
	Social functioning	88.2	19.8	50.0	70.0	100.0	100.0	100.0	
	School functioning	78.2	20.3	40.0	60.0	85.0	100.0	100.0	
	Total PedsQL 4.0	85.7	16.4	59.8	63.0	93.5	100.0	100.0	
Esophageal replacement (n=9)
 
 
 
 	Physical functioning	84.4	19.8	40.6	81.3	90.6	100.0	100.0	
	Emotional functioning	89.8	9.5	70.0	85.0	90.0	95.0	100.0	
	Social functioning	88.9	17.5	50.0	80.0	100.0	100.0	100.0	
	School functioning	75.6	21.9	40.0	55.0	90.0	90.0	100.0	
	Total PedsQL 4.0	84.5	14.3	52.2	84.8	85.9	94.6	98.9	
Primary anastomosis, Gross type C (n=56)
	Physical functioning	87.2	17.5	18.8	78.1	93.8	100.0	100.0	
	Emotional functioning	87.2	14.9	55.0	75.0	92.5	100.0	100.0	
	Social functioning	90.2	14.0	25.0	81.3	95.0	100.0	100.0	
	School functioning	82.3	16.7	50.0	65.0	87.5	95.0	100.0	
	Total PedsQL 4.0	86.6	14.0	42.4	79.3	91.8	97.3	100.0	
Children aged 8-18 years, parent-report									
Delayed primary anastomosis (n=13)	Physical functioning	81.5	24.7	31.3	57.8	100.0	100.0	100.0	
	Emotional functioning	81.9	24.5	30.0	80.0	95.0	100.0	100.0	
	Social functioning	79.6	27.3	20.0	65.0	95.0	100.0	100.0	
	School functioning	68.8	30.5	15.0	55.0	65.0	100.0	100.0	
	Total PedsQL 4.0	79.3	24.3	27.2	69.6	90.2	100.0	100.0	
Esophageal replacement (n=9)	Physical functioning	86.1	15.9	56.3	84.4	87.5	100.0	100.0	
	Emotional functioning	86.1	14.7	60.0	75.0	90.0	100.0	100.0	
	Social functioning	83.3	15.6	60.0	70.0	80.0	100.0	100.0	
	School functioning	76.7	19.2	50.0	60.0	85.0	90.0	100.0	
	Total PedsQL 4.0	83.5	11.3	58.7	79.3	85.9	93.5	95.7	
Primary anastomosis, Gross type C (n=57)	Physical functioning	86.2	19.8	21.9	81.3	93.8	100.0	100.0	
	Emotional functioning	82.7	17.7	30.0	70.0	85.0	100.0	100.0	
	Social functioning	88.8	18.1	18.8	85.0	100.0	100.0	100.0	
	School functioning	79.3	19.7	30.0	70.0	85.0	95.0	100.0	
	Total PedsQL 4.0	84.1	17.5	33.8	80.4	89.1	97.8	100.0	


Condition-specific health-related quality of life scores in children with delayed reconstruction of esophageal atresia (Gross A, B, C) compared to those with primary anastomosis (Gross type C)	
	Scales	Mean	Std Dev	Minimum	Lower Quartile	Median	Upper Quartile	Maximum	
Children aged 2- 7 years, parent-report									
Delayed reconstruction of esophageal atresia (n=8)	Eating	62.2	26.4	20.8	39.3	64.3	82.1	100.0	
	Physical health & treatment	50.8	29.0	16.7	27.1	47.9	69.8	100.0	
	Social isolation & stress	71.6	26.1	43.8	43.8	70.8	100.0	100.0	
	Total EA-QOL	59.6	23.8	33.8	41.4	51.0	82.2	94.1	
Primary anastomosis, esophageal atresia Gross type C (n=22)	Eating	74.8	21.1	29.2	64.3	80.4	89.3	100.0	
	Physical health & treatment	63.1	16.7	33.3	50.0	60.4	70.8	100.0	
	Social isolation & stress	77.4	16.2	50.0	68.8	81.3	87.5	100.0	
	Total EA-QOL	71.6	14.7	43.8	60.3	74.3	80.9	100.0	
Children aged 8-18 years, child-report									
Delayed reconstruction of esophageal atresia (n=20)	Eating	71.7	21.3	17.9	57.8	76.6	84.4	100.0	
	Social relationships	79.1	20.1	32.1	67.9	85.7	96.4	100.0	
	Body perception	90.3	10.4	60.0	85.0	92.5	100.0	100.0	
	Health & well-being	82.2	16.9	43.8	78.1	87.5	93.8	100.0	
	Total EA-QOL	79.0	15.3	45.3	66.7	81.8	92.2	100.0	
Primary anastomosis, esophageal atresia Gross type C (n=31)	Eating	74.9	19.3	34.4	62.5	78.1	90.6	100.0	
	Social relationships	75.3	18.4	42.9	60.7	75.0	89.3	100.0	
	Body perception	81.1	21.6	5.0	80.0	85.0	95.0	100.0	
	Health & well-being	84.3	15.0	31.3	75.0	87.5	93.8	100.0	
	Total EA-QOL	77.8	15.2	34.4	69.8	81.3	87.5	100.0	
Children aged 8-18 years, parent-report									
Delayed reconstruction of esophageal atresia (n=22)	Eating	75.1	22.3	30.0	53.1	82.8	93.8	100.0	
	Social relationships	81.0	20.5	42.9	65.8	88.4	100.0	100.0	
	Body perception	83.3	15.9	50.0	75.0	90.0	100.0	100.0	
	Health & well-being	79.1	16.0	41.7	75.0	81.3	87.5	100.0	
	Total EA-QOL	79.4	16.2	47.9	67.7	80.2	92.7	100.0	
Primary anastomosis, esophageal atresia Gross type C (n=34)	Eating	72.9	21.7	25.0	65.6	75.0	87.5	100.0	
	Social relationships	76.4	17.1	46.4	64.3	78.6	89.3	100.0	
	Body perception	79.1	19.6	5.0	75.0	85.0	90.0	100.0	
	Health & well-being	80.3	18.3	25.0	75.0	87.5	93.8	100.0	
	Total EA-QOL	76.3	16.1	39.6	70.8	82.3	86.5	95.8	
									


Condition-specific health-related quality of life scores in children aged 8-18 years with delayed primary anastomosis, esophageal replacement and those with primary anastomosis, Gross type C	
	Scales	Mean	Std Dev	Minimum	Lower Quartile	Median	Upper Quartile	Maximum	
Children aged 8-18 years, child-report									
Delayed primary anastomosis (n=11)
 
 
 
 	Eating	68.8	19.1	40.6	53.1	75.0	84.4	100.0	
	Social relationships	73.7	23.0	32.1	57.1	78.6	92.9	100.0	
	Body perception	89.5	12.3	60.0	85.0	95.0	100.0	100.0	
	Health & well-being	82.4	18.7	43.8	81.3	87.5	93.8	100.0	
	Total EA-QOL	76.8	15.2	54.2	62.5	78.1	90.6	100.0	
Esophageal replacement (n=9)
 
 
 
 	Eating	75.2	24.4	17.9	75.0	81.3	87.5	100.0	
	Social relationships	86.6	13.2	67.9	73.2	91.1	98.2	100.0	
	Body perception	91.1	8.2	80.0	85.0	90.0	100.0	100.0	
	Health & well-being	81.9	15.5	50.0	75.0	87.5	93.8	100.0	
	Total EA-QOL	81.8	15.9	45.3	79.2	84.4	93.8	95.8	
Primary anastomosis, Gross type C (n=31)
	Eating	74.9	19.3	34.4	62.5	78.1	90.6	100.0	
	Social relationships	75.3	18.4	42.9	60.7	75.0	89.3	100.0	
	Body perception	81.1	21.6	5.0	80.0	85.0	95.0	100.0	
	Health & well-being	84.3	15.0	31.3	75.0	87.5	93.8	100.0	
	Total EA-QOL	77.8	15.2	34.4	69.8	81.3	87.5	100.0	
Children aged 8-18 years, parent-report									
Delayed primary anastomosis (n=11)
 
 
 
 	Eating	70.2	20.4	40.6	50.0	78.1	84.4	100.0	
	Social relationships	76.3	20.0	46.4	57.1	78.6	94.6	100.0	
	Body perception	80.0	16.7	50.0	72.5	77.5	95.0	100.0	
	Health & well-being	79.2	15.8	50.0	68.8	81.3	87.5	100.0	
	Total EA-QOL	76.0	15.3	47.9	66.1	73.9	85.4	100.0	
Esophageal replacement (n=9)
 
 
 
 	Eating	82.2	24.2	30.0	84.4	90.6	96.9	100.0	
	Social relationships	87.9	20.6	42.9	82.1	98.2	100.0	100.0	
	Body perception	87.8	14.6	65.0	80.0	90.0	100.0	100.0	
	Health & well-being	78.9	17.1	41.7	75.0	81.3	87.5	100.0	
	Total EA-QOL	84.0	17.2	55.2	80.2	92.7	94.8	100.0	
Primary anastomosis, Gross type C (n=34)
	Eating	72.9	21.7	25.0	65.6	75.0	87.5	100.0	
	Social relationships	76.4	17.1	46.4	64.3	78.6	89.3	100.0	
	Body perception	79.1	19.6	5.0	75.0	85.0	90.0	100.0	
	Health & well-being	80.3	18.3	25.0	75.0	87.5	93.8	100.0	
	Total EA-QOL	76.3	16.1	39.6	70.8	82.3	86.5	95.8	
									
